# First international descriptive and interventional survey for cholesterol and non-cholesterol sterol determination by gas- and liquid-chromatography–Urgent need for harmonisation of analytical methods

**DOI:** 10.1016/j.jsbmb.2019.03.025

**Published:** 2019-06

**Authors:** Dieter Lütjohann, Ingemar Björkhem, Silvia Friedrichs, Anja Kerksiek, Anita Lövgren-Sandblom, Wolf-Jochen Geilenkeuser, Robert Ahrends, Isabel Andrade, Diana Ansorena, Iciar Astiasarán, Lucía Baila-Rueda, Bianca Barriuso, Susen Becker, Lionel Bretillon, Richard W. Browne, Claudio Caccia, Uta Ceglarek, Ana Cenarro, Peter J. Crick, Günter Fauler, Guadalupe Garcia-Llatas, Robert Gray, William J. Griffiths, Helena Gylling, Scott Harding, Christin Helmschrodt, Luigi Iuliano, Hans-Gerd Janssen, Peter Jones, Leena Kaipiainen, Frank Kannenberg, María Jesús Lagarda, Valerio Leoni, Ana Maria Lottenberg, Dylan S. MacKay, Silke Matysik, Jeff McDonald, Maria Menendez-Carreño, Semone B. Myrie, Valéria Sutti Nunes, Richard E. Ostlund, Eliana Polisecki, Fernando Ramos, Todd C. Rideout, Ernst J. Schaefer, Gerd Schmitz, Yuqin Wang, Chiara Zerbinati, Ulf Diczfalusy, Hans-Frieder Schött

**Affiliations:** a Institute of Clinical Chemistry and Clinical Pharmacology, University Hospital, Bonn, Germany; b Department of Laboratory Medicine, Division of Clinical Chemistry, Karolinska University Hospital, Karolinska Institutet, Huddinge, Sweden; c German Reference Institute for Bioanalytics, Bonn, Germany; d Leibniz-Institut für Analytische Wissenschaften-ISAS-e.V., Dortmund, Germany; e ESTESC-Coimbra Health School, Coimbra, Portugal; f Department of Nutrition, Food Science and Physiology, Faculty of Pharmacy and Nutrition, University of Navarra, Pamplona, Spain; g Hospital Universitario Miguel Servet, IIS Aragón, CIBERV, Zaragoza, Spain; h Institute of Laboratory Medicine, Clinical Chemistry and Molecular Diagnostics, University Hospital Leipzig, Leipzig, Germany; i Department of Pediatric Surgery, University of Leipzig, Leipzig, Germany; j Centre des Sciences du Goŭt et de l'Alimentation, AgroSup Dijon, CNRS, INRA, Université Bourgogne Franche-Comté, Dijon, France; k Biotechnical and Clinical Laboratory Sciences, Jacobs School of Medicine and Biomedical Sciences, University at Buffalo, Buffalo, NY, United States; l Laboratory of Clinical Chemistry, Hospital of Varese, ASST-Settelaghi, Varese, Italy; m Laboratory of Clinical Pathology, Foundation IRCCS Istituto Neurologico Carlo Besta, Milan, Italy; n Institute of Life Science, Swansea University Medical School, Swansea, United Kingdom; o Clinical Institute of Medical and Chemical Laboratory Diagnostics, Medical University of Graz, Graz, Austria; p Nutrition and Food Science Area, University of Valencia, Burjassot, Valencia, Spain; q Department of Nutritional Sciences, Faculty of Life Sciences & Medicine King´s College London, London, UK; r University of Helsinki and Helsinki University Central Hospital, Internal Medicine and Abdominal Center, Helsinki, Finland; s Department of Biochemistry, Faculty of Science, Memorial University of Newfoundland, St. John’s, Canada; t Institute of Pharmacology, Pharmacy and Toxicology VMF, University of Leipzig, Leipzig, Germany; u Department of Medico-Surgical Sciences and Biotechnology, Vascular Biology and Mass Spectrometry Laboratory, Sapienza University of Rome, Latina, Italy; v Unilever Research and Development, Analytical Sciences, Vlaardingen, the Netherlands; w Richardson Centre for Functional Foods and Nutraceuticals, University of Manitoba, Winnipeg, Manitoba, Canada; x Centrum für Laboratoriumsmedizin, Zentrallaboratorium, Universitätsklinikum Münster, Münster, Germany; y Faculty of Medical Sciences, Endocrinology and Metabolism Division, University of Sao Paulo, Sao Paulo, Brazil; z Institute of Clinical Chemistry and Laboratory Medicine, University Hospital Regensburg, Regensburg, Germany; A Department of Molecular Genetics, Southwestern Medical Center, University of Texas, Dallas, United States; B Core Laboratory for Clinical Studies, Division of Endocrinology, Metabolism and Lipid Research, Washington University School of Medicine, St. Louis, MO, 63110, United States; C Boston Heart Diagnostics, Framingham, MA, United States; D REQUIMTE/LAQV, Health Sciences Campus, Faculty of Pharmacy, University of Coimbra, Coimbra, Portugal; E Department of Exercise and Nutrition Sciences, School of Public Health and Health Professions, University of Buffalo, Bufalo, NY, United States

**Keywords:** 5α-chol, 5α-cholestane, epi, epicoprostanol 5α-cholestan-3α-ol, FID, flame ionization detection, GLC, gas-liquid chromatography, LC, liquid chromatography, MSD, mass selective detector, NCS, non-cholesterol sterols, RfB, German Reference Institute for Bioanalytics, Surrogate marker, Cholesterol absorption, Cholesterol synthesis, Cholesterol balance, Atherosclerosis, Phytosterols

## Abstract

Serum concentrations of lathosterol, the plant sterols campesterol and sitosterol and the cholesterol metabolite 5α-cholestanol are widely used as surrogate markers of cholesterol synthesis and absorption, respectively. Increasing numbers of laboratories utilize a broad spectrum of well-established and recently developed methods for the determination of cholesterol and non-cholesterol sterols (NCS). In order to evaluate the quality of these measurements and to identify possible sources of analytical errors our group initiated the first international survey for cholesterol and NCS. The cholesterol and NCS survey was structured as a two-part survey which took place in the years 2013 and 2014. The first survey part was designed as descriptive, providing information about the variation of reported results from different laboratories. A set of two lyophilized pooled sera (A and B) was sent to twenty laboratories specialized in chromatographic lipid analysis. The different sterols were quantified either by gas chromatography-flame ionization detection, gas chromatography- or liquid chromatography-mass selective detection. The participants were requested to determine cholesterol and NCS concentrations in the provided samples as part of their normal laboratory routine. The second part was designed as interventional survey. Twenty-two laboratories agreed to participate and received again two different lyophilized pooled sera (C and D). In contrast to the first international survey, each participant received standard stock solutions with defined concentrations of cholesterol and NCS. The participants were requested to use diluted calibration solutions from the provided standard stock solutions for quantification of cholesterol and NCS. In both surveys, each laboratory used its own internal standard (5α-cholestane, epicoprostanol or deuterium labelled sterols).

Main outcome of the survey was, that unacceptably high interlaboratory variations for cholesterol and NCS concentrations are reported, even when the individual laboratories used the same calibration material. We discuss different sources of errors and recommend all laboratories analysing cholesterol and NCS to participate in regular quality control programs.

## Highlights

• We initiated international surveys for comparison of cholesterol and NCS analysis.
• We are surprised by the very great differences between the laboratories.
• A harmonisation of analytical standard methods is highly needed.

## Graphical abstract

**Figure:**
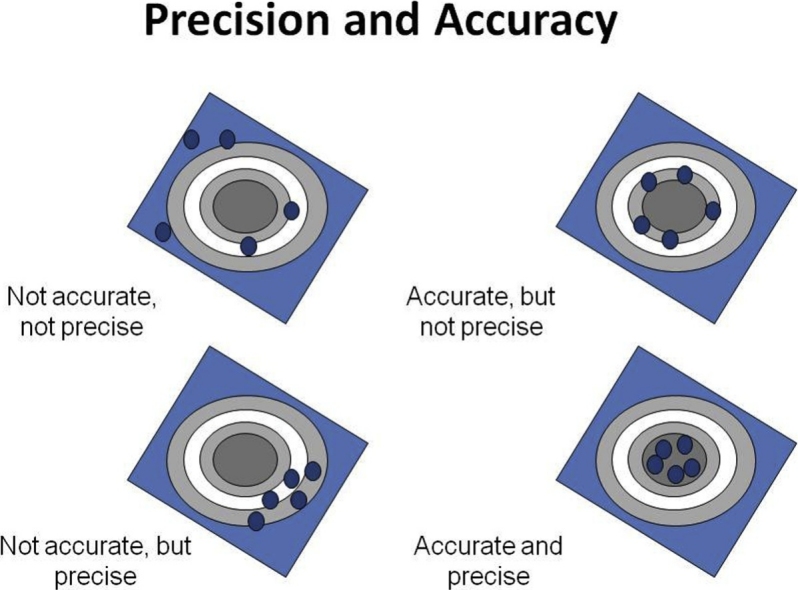

## 1 Introduction

Serum or plasma concentrations of the cholesterol precursors lanosterol, lathosterol, and desmosterol are widely used as surrogate markers of endogenous cholesterol synthesis [1]. The cholesterol metabolite 5α-cholestanol and the plant sterols campesterol and sitosterol are used as markers of cholesterol absorption [2,3]. These non-cholesterol sterols (NCS) show even stronger correlations with cholesterol absorption and synthesis when expressed as ratios to total cholesterol, which standardizes for variations in sterol transport protein concentrations [4]. Specifically, when reporting NCS as their ratios to cholesterol, the cholesterol measurement should ideally be performed from the same sample preparation as for the NCS analysis. The interest in surrogate markers of cholesterol metabolism is recently increasing and more laboratories develop new methods for the determination of cholesterol and NCS using different chromatographic separation and mass spectrometric detection methods. Conflicting absolute and cholesterol-corrected NCS concentrations are reported in the literature making it difficult or nearly impossible to define cut-off values [[5], [6], [7]] or to compare absolute or cholesterol corrected values from different studies in meta-analysis. For a better comparison of reported values and the identification of methodological sources of errors, we planned and performed the first international surveys for cholesterol and NCS. The first part of the cholesterol and NCS survey was initiated in the year 2013 under the expertise of the German Reference Institute for Bioanalytics (RfB) and the Laboratory for Specialized Lipid Diagnostic of the University Hospital, both located in Bonn, Germany. The aim of the first survey was to investigate the variations in cholesterol and NCS concentrations routinely determined by different laboratories in Europe, North and South America. Here, we reflect the comparability of cholesterol and NCS concentrations determined by different separation and detection methods and discuss the suitability of different compounds used as internal standards for sterol quantification. Twenty laboratories specialized in chromatographic lipid analysis, by either gas- or liquid-chromatography were enrolled to participate in this first survey part. The second part of the international cholesterol and neutral sterol survey took place one year later in 2014. Twenty-two laboratories participated in the second survey whereof five laboratories attended for the first time. Contrary to the first survey, the second was designed as an interventional trial. The focus was on the influence of the utilized calibration solutions and the participants were requested to use provided stock solutions for the quantification of the sample material.

## 2 Materials and methods

**Fig. 1:**
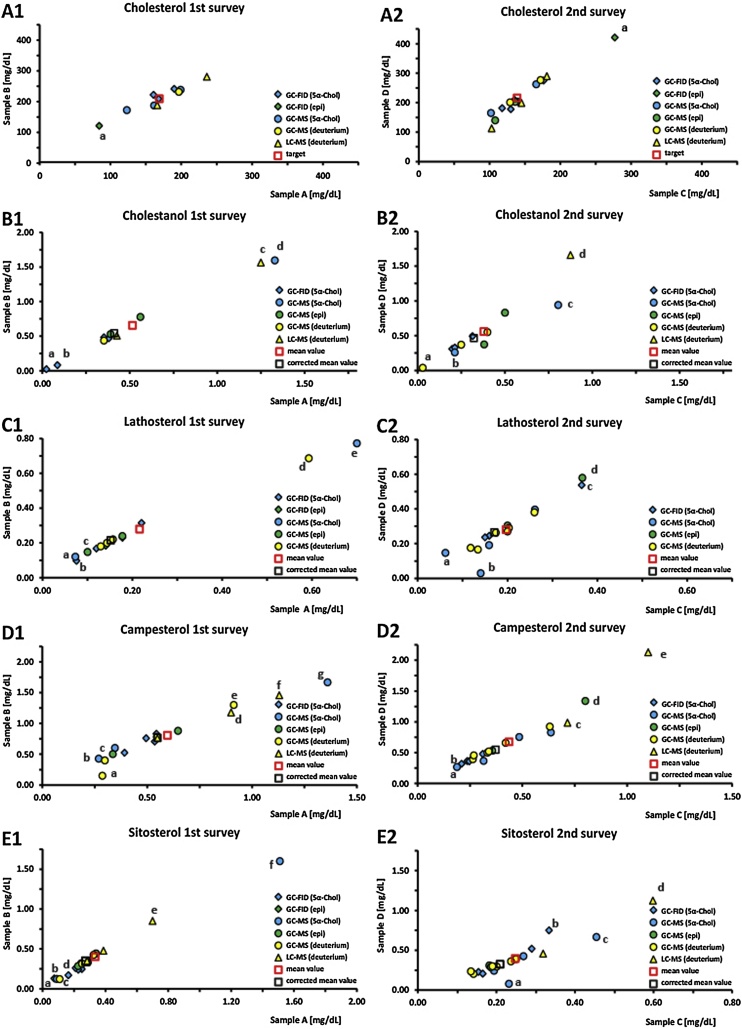
Distribution of absolute cholesterol and non-cholesterol sterol concentrations analyzed by GC–MS and LC–MS methods in the first descriptive and second interventional survey. Values a–e in the figures A1-E2 are regarded as outliers.

**Fig. 2:**
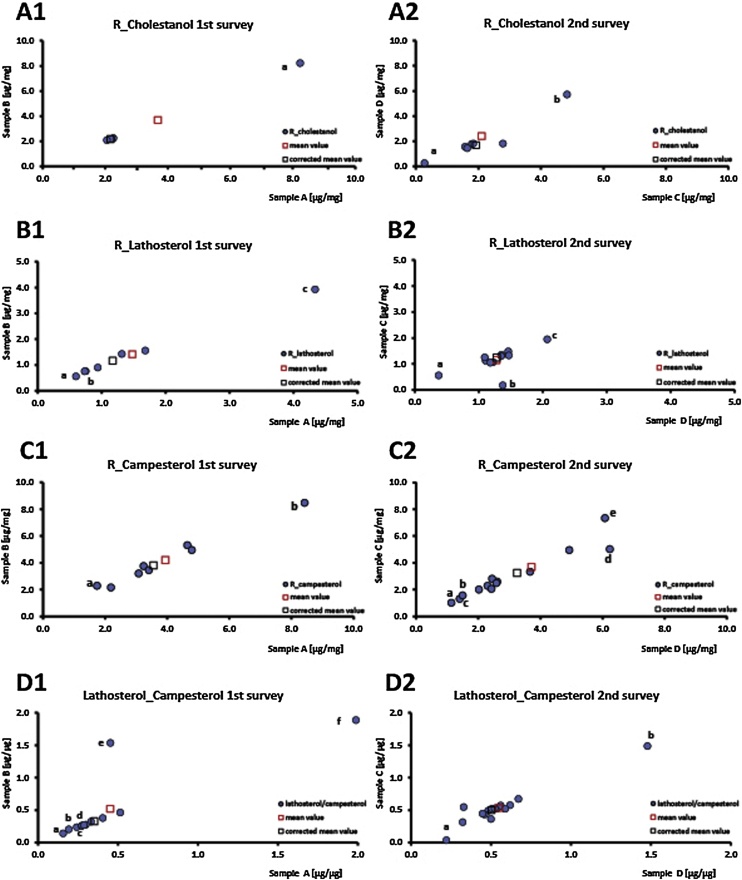
Distribution of calculated cholesterol corrected non-cholesterol concentrations and ratio lathosterol to campesterol in the first descriptive and second interventional survey. Values a–e in the figures A1-D2 are regarded as outliers.

The participants submitted results from six different methods for sterol determination: capillary gas chromatography-flame ionisation detection (GC-FID) with either 5α-cholestane (5α-chol) or epicoprostanol (epi) as internal standard (GC-FID-5α-chol and GC-FID-epi, respectively), capillary GC-mass selective detection (MSD) with either 5α-chol, epi or deuterium labelled sterols as internal standards (GC-MSD-5α-chol, GC-MSD-epi and GC-MSD-deuterium, respectively) and high performance-liquid chromatography (HPLC) with MSD and deuterated sterols (LC-MSD-deuterium) used as internal standard. Since each laboratory was requested to use its specific routine analytical method, the work-up procedure and determination setting are strikingly different for the individual participants. Table 1 shows an overview of the reported sample work-up conditions, chemicals, chromatographic columns, and instrumentation for GC analyses used in the surveys. Most of the laboratories used alkaline hydrolysis in order to deconjugate fatty acid esterified sterols and thus analysing total serum sterol concentrations after derivatisation of the free hydroxyl groups. One laboratory, using LC-MSD, quantified free and esterified fraction of the freeze-dried test-samples in order to calculate the total concentration. Some laboratories used fully automated peak integration and quantitation software belonging to the software package of the supplier. Others integrated the individual peaks half-automatically using self-created integration and evaluation macros with the option to supervise and correct integration of each peak. It should be emphasized that the individual sample work-up procedures or detection specifications cannot be reported within this article because of the anonymization of the participants. Since the freeze-dried serum samples were routinely used in round-robin tests of the RfB, it was possible to give a reference value (target concentration) only for cholesterol. The lyophilized serum samples were additionally used to measure other parameters in clinical routine biochemistry such as triglycerides, total protein or subspecies proteins such as albumin or gamma globuline in additional ring trials in Germany. All these parameters were within the normal range as characterized by reference values except for triglycerides in sample D of the second ring trial, where the median level was marginally increased (data not shown). Single values more than 50% different from the mean value of all submitted sterol concentrations have a marked effect on the mean values of the whole participant group. Therefore, individual values more than 50% different from the mean value were regarded as outliers and several of the above-mentioned parameters were additionally calculated without the outlier values and reported within the script. The guidelines of the German Medical Association for quality assurance of clinical laboratory investigations tolerates relative deviations for cholesterol within external proficiency testing of up to 13% [8]. Due to the relatively small number of participants in our surveys we set a relative deviation, that can be considered as acceptable to ± 15% of the mean value of the included participants. The mean values, standard deviation, minimum and maximum values of all participants as well as the ratios of 5α-cholestanol, lathosterol, campesterol to cholesterol and the ratio lathosterol to campesterol are listed in Table 2, Table 3 and shown in Fig. 1, Fig. 2.

**Table 1:** Methodological parameters and instrumental equipment from the different contributors.

sample volume [μL]	10; 20; 40; 50; 100; 200; 250; 300; 400; 500; 1000
hydrolysis time [min]	15; 20; 60; 65; 68; 70; 90; 120
hydrolysis temperature [°C]	50; 60; 80; 90
hydrolysis NaOH/KOH concentration [mol/L]	0.35; 0.5; 0.71; 1.0; 1.78; 2.0
extraction solvents	hexane; petroleum ether; petroleum ethyl ether; chloroforme; tetrachlorethylene; cyclohexane; isooctane; heptane
solvent volume for extraction [mL]	2; 3; 4; 5; 9; 20
silylation time [min]	30; 45; 60; over night
silylation temperature [°C]	37; 55; 60; 70
silylation reagents (GC only)	HMDS/TMCS/pyridine (3:1:9)(Supelco) dimethylformamide/HMDS/TMCS Sylon BFT BSTFA/TMCS 99:1 + pyridine TriSil reag (Thermo Fischer) HMDS/TMCS/Pyridine 2:1:3 MSTFA
Injection volume [μL]	1; 2; 3; 25; 40
split mode injection (GC only)	1:3; 10:1; 12:1; 15:1; 20:1; splitless
column lenght (GC only) [m]	25; 30
column diameter (GC only) [mm]	0.22; 0.25; 0.32
column film thickness (GC only) [μm]	0.1; 0.25
columns (GC only)	SUPELCO SAC™-5 Capillary GC Column HP-Ultra2 SAC-5 capilary column DB-5-MS Restek RXI 1 mS (100% dimethyl polysiloxane) CP_SIL_5CB HAT-5 carbone modified siloxane HP-5MS 5% phenylmethyl siloxane
separation instruments (GC only)	GC-Agilent Technologies 6890 N GC-Agilent 7890A GC-Shimazu GC-17A FID GC-HP4890 Shimadzu GC-MSQP2010
detectors	Flame ionisation detector Mass selective detector 5975c inert xl ms Ion Trap AgilentMS 5973 N 4000 Qtrap triple quad SCIEX API 3000 triple quadrupol Photospray Thermo TSQ8000 Triple quad

**Table 2:** Results of absolute cholesterol and non-cholesterol sterols submitted by GC–MS and LC–MS methods.

	First survey								Second survey								Participants		
	Sample A				Sample B				Sample C				Sample D				1st survey	2nd survey	both surveys
	mean ± SD^1)^	CV^2)^	min^3)^	max^4)^	mean ± SD^1)^	CV^2)^	min^3)^	max^4)^	mean ± SD^1)^	CV^2)^	min^3)^	max^4)^	mean ± SD^1)^	CV^2)^	min^3)^	max^4)^			
*total cholesterol*	168±42	25.1%	84	236	211±45	21.6%	118	285	147±43	28.9%	102	277	220±73	33.0%	113	421	10	16	8
*GC-FID (5α-cholestane)*	173±15	8.8%	161	190	224±16	7.3%	208	241	140±20	14.2%	118	176	209±35	16.8%	178	275	3	6	3
*GC-FID (epicoprostanol)*	84				118				277				421				1	1	1
*GC-MS (5α-cholestane)*	161±38	23.6%	123	199	203±35	17.0%	176	242	134±45	33.8%	102	166	214±69	32.2%	165	262	3	2	1
*GC-MS (epicoprostanol)*									122±20	16.3%	108	136	171±44	25.8%	140	203		2	
*GC-MS (deuterium)*	196				235				151±30	20.2%	129	172	239±54	22.4%	201	277	1	2	1
*LC-MS (deuterium)*	201±49	24.6%	166	236	238±65	27.5%	192	285	143±39	27.3%	103	181	201±89	44.1%	113	290	2	3	2
***Target5)***	**169**				**219**				**139**				**216**						

***total cholestanol***	**0.51±0.44**	**85.1%**	**0.02**	**1.33**	**0.63±0.53**	**83.9%**	**0.02**	**1.57**	**0.38±0.26**	**68.3%**	**0.03**	**0.88**	**0.56±0.45**	**79.6%**	**0.04**	**1.66**	**10**	**11**	**7**
*GC-FID (5α-cholestane)*	0.21±0.18	86.6%	0.02	0.38	0.26±0.25	93.2%	0.02	0.48	0.24±0.06	26.3%	0.20	0.32	0.38±0.10	26.7%	0.31	0.49	4	3	2
*GC-MS (5α-cholestane)*	1.33				1.57				0.51±0.42	82.2%	0.21	0.81	0.60±0.48	80.4%	0.26	0.94	1	2	1
*GC-MS (epicoprostanol)*	0.48±0.12	25.4%	0.39	0.56	0.63±0.18	28.1%	0.50	0.75	0.44±0.08	19.2%	0.38	0.50	0.60±0.32	53.6%	0.37	0.83	2	2	2
*GC-MS (deuterium)*	0.35				0.41				0.23±0.19	82.3%	0.03	0.40	0.32±0.26	81.3%	0.04	0.55	1	3	1
*LC-MS (deuterium)*	0.84±0.58	69.5%	0.43	1.25	1.01±0.75	73.9%	0.48	1.54	0.88				1.66				2	1	1

***total lathosterol***	**0.22±0.20**	**91.0%**	**0.07**	**0.70**	**0.26±0.21**	**80.5%**	**0.10**	**0.75**	**0.20±0.09**	**43.5%**	**0.06**	**0.37**	**0.28±0.15**	**51.8%**	**0.03**	**0.58**	**13**	**15**	**10**
*GC-FID (5α-cholestane)*	0.15±0.06	42.9%	0.08	0.22	0.19±0.08	43.3%	0.10	0.30	0.22±0.12	54.0%	0.15	0.37	0.34±0.17	50.5%	0.24	0.54	4	3	2
*GC-FID (epicoprostanol)*	0.14				0.18												1		
*GC-MS (5α-cholestane)*	0.39±0.44	114.6%	0.07	0.70	0.42±0.46	108.7%	0.10	0.75	0.16±0.08	52.7%	0.06	0.26	0.19±0.15	80.1%	0.03	0.40	2	4	2
*GC-MS (epicoprostanol)*	0.14±0.05	39.4%	0.10	0.18	0.17±0.06	37.8%	0.13	0.22	0.28±0.12	41.6%	0.20	0.37	0.44±0.19	44.0%	0.30	0.58	2	2	2
*GC-MS (deuterium)*	0.26±0.23	88.0%	0.13	0.59	0.30±0.24	81.4%	0.16	0.66	0.18±0.05	28.4%	0.12	0.26	0.26±0.08	30.8%	0.17	0.38	4	6	4

***total campesterol***	**0.60±0.32**	**53.8%**	**0.27**	**1.36**	**0.76±0.41**	**54.0%**	**0.10**	**1.62**	**0.44±0.25**	**56.5%**	**0.19**	**1.10**	**0.68±0.46**	**67.7%**	**0.27**	**2.13**	**16**	**18**	**12**
*GC-FID (5α-cholestane)*	0.49±0.07	14.2%	0.39	0.54	0.66±0.13	20.1%	0.47	0.78	0.25±0.04	17.0%	0.21	0.31	0.38±0.07	17.9%	0.32	0.48	4	4	2
*GC-MS (5α-cholestane)*	0.66±0.61	92.5%	0.27	1.36	0.85±0.67	78.9%	0.38	1.62	0.41±0.20	48.2%	0.19	0.64	0.55±0.28	50.2%	0.27	0.83	3	4	2
*GC-MS (epicoprostanol)*	0.49±0.22	44.7%	0.34	0.65	0.64±0.27	41.6%	0.45	0.83	0.58±0.31	54.1%	0.36	0.80	0.94±0.57	60.9%	0.53	1.34	2	2	2
*GC-MS (deuterium)*	0.51±0.29	57.5%	0.29	0.91	0.61±0.50	82.0%	0.10	1.25	0.38±0.14	36.5%	0.26	0.63	0.58±0.19	33.4%	0.39	0.92	4	6	4
*LC-MS (deuterium)*	0.86±0.29	34.0%	0.55	1.13	1.08±0.35	32.4%	0.71	1.41	0.91±0.27	30.0%	0.72	1.10	1.56±0.81	51.9%	0.99	2.13	3	2	2

***total sitosterol***	**0.33±0.33**	**100.2%**	**0.07**	**1.51**	**0.40±0.35**	**88.0%**	**0.12**	**1.60**	**0.25±0.12**	**46.8%**	**0.14**	**0.60**	**0.39±0.24**	**61.3%**	**0.08**	**1.10**	**17**	**19**	**12**
*GC-FID (5α-cholestane)*	0.21±0.04	17.1%	0.16	0.25	0.27±0.04	15.2%	0.21	0.30	0.24±0.09	35.6%	0.15	0.33	0.40±0.24	58.4%	0.21	0.75	4	5	2
*GC-FID (epicoprostanol)*	0.07				0.13												1		
*GC-MS (5α-cholestane)*	0.62±0.78	126.6%	0.09	1.51	0.68±0.80	118.0%	0.12	1.60	0.29±0.12	40.2%	0.19	0.45	0.35±0.25	71.1%	0.08	0.67	3	4	2
*GC-MS (epicoprostanol)*	0.28±0.08	28.6%	0.23	0.34	0.36±0.11	30.9%	0.28	0.44	0.18±0.00	1.5%	0.18	0.19	0.30±0.01	4.8%	0.29	0.31	2	2	2
*GC-MS (deuterium)*	0.24±0.09	38.9%	0.11	0.32	0.30±0.13	43.0%	0.12	0.42	0.19±0.05	24.6%	0.14	0.25	0.30±0.07	23.8%	0.20	0.39	4	6	4
*LC-MS (deuterium)*	0.46±0.22	47.5%	0.28	0.70	0.56±0.26	46.6%	0.35	0.85	0.46±0.20	43.3%	0.32	0.60	0.78±0.45	58.1%	0.46	1.10	3	2	2

^1)^ mean value with standard deviation [mg/dL], ^2)^ coefficient of variation, ^3)^ minimum value [mg/dL], ^4)^ maximum value [mg/dL], ^5)^ determined by reference institute.

**Table 3:** Results of cholesterol corrected non-cholesterol sterols and ratio lathosterol to campesterol submitted by GC–MS and LC–MS methods.

H		First survey								Second survey								Participants		
		Sample A				Sample B				Sample C				Sample D				1st survey	2nd survey	**both surveys**
		mean ± SD^1)^	CV^2)^	min^3)^	max^4)^	mean ± SD^1)^	CV^2)^	min^3)^	max^4)^	mean ± SD^1)^	CV^2)^	min^3)^	max^4)^	mean ± SD^1)^	CV^2)^	min^3)^	max^4)^			
***total ratio cholestanol to***	***cholesterol5)***	**3.68±3.04**	**82.4%**	**2.05**	**8.24**	**3.68±3.02**	**82.1%**	**2.09**	**8.22**	**2.10±1.41**	**67.1%**	**0.27**	**4.83**	**2.41±2.33**	**96.5%**	**0.27**	**5.72**	**4**	**7**	**2**
*GC-FID (5α-cholestane)*	*GC-FID (5α-cholestane)*	2.23±0.05	2.2%			2.22±0.05	2.3%			1.68±0.15	9.0%			1.68±0.16	9.3%			2	2	1
*GC-MS (5α-cholestane)*	*GC-FID (5α-cholestane)*									1.64				1.46					1	
*GC-MS (epicoprostanol)*	*GC-FID (5α-cholestane)*	2.05				2.09				2.78				1.82				1	1	
*GC-MS (5α-cholestane)*	*GC-MS (5α-cholestane)*	8.24				8.22												1		
*GC-MS (deuterium)*	*GC-MS (epicoprostanol)*									1.05±1.05	99.5%			1.05±1.10	104.8%				2	
*LC-MS (deuterium)*	*LC-MS (deuterium)*									4.83				5.72					1	

***total ratio lathosterol to***	***cholesterol5)***	**1.48±1.32**	**89.1%**	**0.60**	**4.33**	**1.41±1.17**	**82.8%**	**0.56**	**3.93**	**1.28±0.40**	**31.4%**	**0.37**	**2.07**	**1.15±0.47**	**40.5%**	**0.18**	**1.95**	**7**	**11**	**5**
*GC-FID (5α-cholestane)*	*GC-FID (5α-cholestane)*	1.03±0.40	38.9%			1.09±0.47	43.2%			1.51±0.50	33.1%			1.48±0.43	28.9%			2	3	2
*GC-MS (5α-cholestane)*	*GC-FID (5α-cholestane)*									1.22				1.07					1	
*GC-MS (epicoprostanol)*	*GC-FID (5α-cholestane)*	0.94				0.90				1.46				1.48				1	1	1
*GC-FID (epicoprostanol)*	*GC-FID (epicoprostanol)*	1.68				1.56												1		
*GC-MS (5α-cholestane)*	*GC-MS (5α-cholestane)*	2.46**±**2.64	107.3%			2.24**±**2.38	106.3%			0.87**±**0.71	81.2%			0.37**±**0.27	72.9%			2	2	1
*GC-MS (deuterium)*	*GC-MS (epicoprostanol)*									1.28**±**0.27	20.8%			1.29**±**0.06	4.3%				2	
*GC-MS (deuterium)*	*GC-MS (deuterium)*	0.73				0.75				1.26**±**0.12	9.4%			1.18**±**0.18	15.5%			1	2	1

***total ratio campesterol to***	***cholesterol5)***	**3.94±2.10**	**53.2%**	**1.74**	**8.42**	**4.20±2.06**	**49.0%**	**2.16**	**8.48**	**3.71±1.86**	**50.1%**	**1.13**	**6.23**	**3.70±1.98**	**53.5%**	**1.02**	**7.34**	**8**	**13**	**6**
*GC-FID (5α-cholestane)*	*GC-FID (5α-cholestane)*	3.16±0.11	3.6%			3.48±0.39	11.2%			1.80±0.43	23.7%			1.79±0.44	24.3%			2	4	2
*GC-MS (5α-cholestane)*	*GC-FID (5α-cholestane)*									2.42				2.06					1	
*GC-MS (epicoprostanol)*	*GC-FID (5α-cholestane)*	3.41				3.45				2.61				2.60				1	1	1
*GC-MS (5α-cholestane)*	*GC-MS (5α-cholestane)*	4.12**±**3.74	90.8%			4.31**±**3.61	83.8%			3.68**±**3.61	97.9%			3.02**±**2.83	93.7%			3	2	1
*GC-MS (deuterium)*	*GC-MS (epicoprostanol)*									2.44				2.81					1	
*GC-MS (deuterium)*	*GC-MS (deuterium)*	4.65				5.32				3.12**±**0.77	24.6%			2.91**±**0.60	20.7%			1	2	1
*LC-MS (deuterium)*	*LC-MS (deuterium)*	4.79				4.95				5.50**±**0.81	14.8%			6.15**±**1.69	27.5%			1	2	1

***total ratio lathosterol to***	***campesterol6)***	**0.45±0.49**	**109.6%**	**0.16**	**1.99**	**0.52±0.57**	**109.2%**	**0.14**	**1.89**	**0.55±0.28**	**51.5%**	**0.22**	**1.48**	**0.54±0.30**	**56.4%**	**0.04**	**1.49**	**12**	**15**	**10**
*GC-FID (5α-cholestane)*	*GC-FID (5α-cholestane)*	0.29±0.09	32.2%			0.29±0.08	28.0%			0.88±0.53	60.3%			0.88±0.53	59.9%			4	3	2
*GC-MS (5α-cholestane)*	*GC-MS (5α-cholestane)*	0.39±0.17	43.6%			0.36±0.14	40.2%			0.40±0.15	37.7%			0.41±0.25	60.9%			2	4	2
*GC-MS (epicoprostanol)*	*GC-MS (epicoprostanol)*	0.29±0.02	5.8%			0.27±0.01	4.1%			0.51±0.07	14.1%			0.50±0.10	19.5%			2	2	2
*GC-MS (deuterium)*	*GC-MS (deuterium)*	0.72±0.85	118.3%			0.96±0.88	92.1%			0.50±0.11	21.4%			0.46±0.10	22.3%			4	6	4

^1)^ mean value with standard deviation, ^2)^ coefficient of variation, ^3)^ minimum value, ^4)^ maximum value, ^5)^ [μg/mg], ^6)^ [μg/μg].

### 2.1 The first international cholesterol and NCS survey

Twenty laboratories specialized in chromatographic lipid analysis participated in this survey. A set of two different lyophilized pooled sera (sample A and B) was sent to each participant and analysed with the individual determination method during the routine operation of each laboratory. After a period of twelve weeks the participants were requested to submit their results to RfB for further data analysis. Eighteen of the twenty participating laboratories submitted their results before the 12-week deadline and were included for further evaluation. The data were submitted in the units individually used by the participant (ng/mL, mg/mL, μg/dL, mg/dL, mg/L, μmol/L, mmol/L) and converted into standard units for comparison (cholesterol and NCS as mg/dL). Each participant received the results presented in form of Youden-Plots and basic statistical data. The evaluated data were given in the individual units, which were used by the participant.

### 2.2 The second international cholesterol and NCS survey

Twenty-two laboratories participated in the second survey whereof the results of five laboratories were included that attended for the first time. Two groups that attended the first survey were not able to participate in the second survey. Again a set of two different lyophilized pooled sera (sample C and D) was sent to each participant and analysed with the individual determination method used in the routine operation of each laboratory. Contrary to the first survey the second part was designed as an interventional trial with focus on the influence of the utilized calibration solutions on cholesterol and NCS concentrations. Therefore, the participants were requested to use the in glass ampoules provided stock solutions (containing cholesterol, 5α-cholestanol, lathosterol, campesterol, and sitosterol in the concentrations 1.0 mg/mL; 18.8 μg/mL, 18.8 μg/mL, 13.0 μg/mL, and 18.0 μg/mL; respectively). The stock solutions for the quantification of the sample material were generously provided by the Department of Laboratory Medicine, Division of Clinical Chemistry, Karolinska University Hospital, Karolinska Institutet, Huddinge, Sweden. All non-cholesterol sterols for the quantification of the sample material were purchased from Sigma-Aldrich. Data collection and result transmission procedure was identically to the above described protocol of the first survey part.

### 2.3 Statistical analysis

Statistical parameters (mean values, standard deviations, coefficients of variation, maximum and minimum values) of the submitted concentrations of all participating laboratories were calculated. Conditions that impede a clear and strong statistical evaluation of the data set are the fact that different laboratories participated in the two surveys (only twelve laboratories participated in both surveys). For that reason, only a qualitative discussion of the data set is performed.

## 3 Results and discussion

The results and subgroup analysis of cholesterol and NCS from the both surveys are listed in Table 2 and shown in Fig. 1, A1 to E2.

### 3.1 Cholesterol

In the first survey for chromatographically determined cholesterol ten different laboratories participated. The mean cholesterol concentrations determined by all ten participants were calculated for sample A (168 ± 42 mg/dL; CV 25.1%) and B (211 ± 45 mg/dL; CV 21.6%). The target concentrations specified by the RfB were 169 mg/dL (sample A) and 219 mg/dL (sample B). Five out of ten participants did not determine cholesterol concentrations within a ± 15% range of the target value. One participant was identified as outlier (Fig. 1A1, a).

In the second survey part sixteen different laboratories attended from which eight participated in both surveys. The mean cholesterol concentrations determined by all sixteen participants were calculated for sample C (147 ± 43 mg/dL; CV 28.9%) and D (220 ± 73 mg/dL; CV 33.0%). The target concentrations specified by the reference institute were 139 mg/dL (sample C) and 216 mg/dL (sample D). Only six out of sixteen participants reported cholesterol values that are within a ± 15% range of our accepted target value. One participant was identified as outlier (Fig. 1A2, a).

The differences in reported cholesterol results are difficult for an interpretation. Probably insufficient fatty acid deconjugation of the cholesterol esters during alkaline hydrolysis might have impaired the analysis of some participants. It is noteworthy that even with methods based on isotope dilution GC-MSD and use of an deuterated internal standard and the same calibration material two laboratories reported a difference of about 25%. Since our survey shows that two of the three laboratories using GC-FID reported excellent results, the present data does not enable the conclusion that deuterium labelled cholesterol used as internal standard is superior to the use of 5α-cholestane or epicoprostanol. The greatest variations in cholesterol determination were obtained using methods based on LC-MSD.

### 3.2 5α-cholestanol

In the first survey for chromatographic analysis of serum 5α-cholestanol ten different laboratories participated. The mean 5α-cholestanol concentration determined by all ten participants were calculated for sample A (0.515 ± 0.438 mg/dL; CV 85.1%) and B (0.632 ± 0.530 mg/dL; CV 83.9%). Therefore, none of the participants determined 5α-cholestanol concentrations within a ± 15% range of the mean value calculated of the reported of all participants. The concentrations submitted by four out of ten laboratories were identified as outliers (Fig. 1 B1, a to d) and new corrected mean concentrations for the remaining six laboratories were calculated (sample A: 0.410 ± 0.079 mg/dL, CV 19.2%; sample B: 0.517 ± 0.120 mg/dL, CV 23.1%). Out of these remaining six laboratories, four determined 5α-cholestanol concentrations within a ± 15% range of the corrected mean value.

In the second survey eleven different laboratories attended from which seven laboratories participated in both surveys. The mean 5α-cholestanol concentrations determined by eleven participants were calculated for sample C (0.380 ± 0.260 mg/dL; CV 68.3%) and D (0.559 ± 0.445 mg/dL; CV 79.6%). One of the participants determined 5α-cholestanol concentrations within a ± 15% range of the mean value of all participants and four participants reported results which are identified as outliers (Fig. 1 B2, a to d). The new corrected mean concentration for the remaining seven laboratories was calculated as (sample C: 0.322 ± 0.111 mg/dL, CV 34.4%; sample D: 0.465 ± 0.183 mg/dL, CV 39.4%). Only one out of the seven remaining laboratories reported results within a ± 15% range of the corrected mean value.

The first survey revealed a 20-fold variation in the individual results between the highest and lowest reported concentration. Also, the second survey revealed widely scattered concentrations with four distinct outliers. Even after the exclusion of these outliers, a 2.5 fold variation between the highest and lowest included concentrations persisted in the second survey. One of the major problems with the 5α-cholestanol determination may be related to the fact that 5α-cholestanol has very similar chromatographic properties to cholesterol, whose concentration in serum are 300 fold higher. Therefore, the separation performance of the chromatographic system may be a critical for a reliable 5α-cholestanol quantification.

### 3.3 Lathosterol

In the first part of the survey for assay of lathosterol thirteen different laboratories participated. The mean lathosterol concentrations determined by all thirteen participants were calculated for sample A (0.216 ± 0.197 mg/dL; CV 91.0%) and B (0.257 ± 0.207 mg/dL; CV 80.5%). None of the participants reported lathosterol levels within a ± 15% range of the mean value of all participants. The concentrations submitted by five of the thirteen laboratories were regarded as outliers (Fig. 1 C1, a and e) and new, corrected mean concentrations for the remaining eleven laboratories were calculated (sample A: 0.158 ± 0.033 mg/dL, CV 20.6%; sample B: 0.201 ± 0.044 mg/dL, CV 21.7%). After the correction for the outlier values five of the remaining eleven laboratories reported lathosterol concentrations within a ± 15% range of the corrected mean value.

In the second survey fifteen different laboratories attended from which ten laboratories participated in both surveys. The mean lathosterol concentration obtained from all fifteen participants were calculated for sample C (0.197 ± 0.086 mg/dL; CV 43.5%) and D (0.281 ± 0.145 mg/dL; CV 51.8%). Four of the participants determined lathosterol concentrations within a ± 15% range of the mean value of all participants. In the second survey, four participants were identified as outliers (Fig. 1 C2, a to d) and new corrected mean concentrations for the remaining eleven laboratories were calculated (sample C: 0.183 ± 0.047 mg/dL, CV 25.5%; sample D: 0.265 ± 0.076 mg/dL, CV 28.5%). Only five of the remaining laboratories reported results within a ± 15% range of the common mean value.

In both surveys very high variations of lathosterol concentrations were reported. After exclusion of 4 outliers in the second survey a 2.0 fold variation between the reported results remained.

While chromatographic separation of 5α-cholestanol in presence of high serum cholesterol concentrations is very challanging, lathosterol can be chromatographically well separated from other non-cholesterol sterols. However, incorrect peak identification and/or incorrect integration of the peak could lead to these discrepancies in the results reported from the different laboratories.

### 3.4 Campesterol

In the first survey for chromatographically determined campesterol sixteen different laboratories participated. The mean campesterol concentrations determined by all sixteen participants were calculated for sample A (0.597 ± 0.321 mg/dL; CV 53.8%) and B (0.760 ± 0.410 mg/dL; CV 54.0%). Five of the participants reported campesterol concentrations within a ± 15% range of the mean value of all participants. The concentrations reported by seven of the sixteen laboratories were identified as outliers (Fig. 1 D1, a to g) and new corrected mean concentrations for the remaining eleven laboratories were calculated (sample A: 0.488 ± 0.106 mg/dL, CV 21.8%; sample B: 0.656 ± 0.134 mg/dL, CV 20.4%). Four of these remaining nine laboratories determined campesterol concentrations within a ± 15% range of the corrected mean value.

In the second survey, eighteen different laboratories participated from which twelve laboratories participated in both surveys. The mean campesterol concentrations determined by all eighteen participants were calculated as for sample C (0.437 ± 0.247 mg/dL; CV 56.5%) and D (0.677 ± 0.458 mg/dL; CV 67.7%). Two of the eighteen participants determined campesterol concentrations within a ± 15% range of the mean value of all participants. In the second survey five participants were regarded as outliers (Fig. 1 D2, a to e) and corrected mean concentrations for the remaining thirteen laboratories were calculated (sample C: 0.373 ± 0.135 mg/dL, CV 36.1%; sample D: 0.549 ± 0.187 mg/dL, CV 33.9%). Only three of the remaining laboratories determined results within an ± 15% range of the corrected mean value.

For the measurement of campesterol very high variations were reported in both surveys. After exclusion of 5 outliers in the second survey there was still a 2.5 fold difference between the highest and lowest reported value.

### 3.5 Sitosterol

In the first survey part for chromatographically determined sitosterol seventeen different laboratories participated. The mean sitosterol concentrations determined by all seventeen participants were calculated for sample A (0.334 ± 0.335 mg/dL; CV 100.2%) and B (0.401 ± 0.353 mg/dL; CV 88.0%). Two of the participants determined sitosterol concentrations within a ± 15% range of the mean value of all participants. The concentrations submitted by six of the seventeen laboratories were identified as outliers (Fig. 1 E1, a to f) and new corrected mean concentrations for the remaining eleven laboratories were calculated (sample A: 0.276 ± 0.055 mg/dL, CV 20.1%; sample B: 0.345 ± 0.071 mg/dL, CV 20.5%). Of the remaining eleven laboratories four determined sitosterol concentrations within a ± 15% range of the corrected mean value.

In the second survey, nineteen different laboratories participated from which twelve laboratories participated in both surveys. The mean sitosterol concentrations determined by all eighteen participants were calculated for sample C (0.248 ± 0.116 mg/dL; CV 46.8%) and D (0.387 ± 0.237 mg/dL; CV 61.3%). Only three of the nineteen participants reported sitosterol concentrations within a ± 15% range of the mean value of all participants. In the second survey four participants were identified as outliers (Fig. 1 E2, a to d) and a corrected mean concentrations for the remaining sixteen laboratories were calculated (sample C: 0.206 ± 0.055 mg/dL, CV 26.6%; sample D: 0.317 ± 0.095 mg/dL, CV 30.1%). Only six of the remaining laboratories determined results within a ± 15% range of the corrected mean value.

Also, in both surveys a high variation in the reported sitosterol concentrations was observed. After removing 4 outliers in the second survey there was still a 2.0 fold difference between the highest and the lowest reported value.

### 3.6 Ratios of 5α-cholestanol, lathosterol, and campesterol to cholesterol and lathosterol to campesterol

Since the ratios of 5α-cholestanol, lathosterol, and campesterol to cholesterol and lathosterol to campesterol are the most common surrogate markers for absorption and synthesis rates, we calculated these ratios for sample A to D for all participants. The results and subgroup analysis from the both surveys are listed in details in Table 2 and shown in Fig. 2 A1 to D2.

#### 3.6.1 5α-cholestanol to cholesterol

In the first survey part four participants were included with calculated results for sample A (3.68 ± 3.04 μg/mg; CV 82.4%) and B (3.68 ± 3.02 μg/mg; CV 82.1%). None of the participants determined 5α-cholestanol to cholesterol ratio within a ± 15% range of the mean value of all participants and one participant was identified as outlier (Fig. 2 A1, a). The new corrected mean value for the remaining three laboratories were calculated for A (2.17 ± 0.11 μg/mg; CV 4.9%) and B (2.17 ± 0.08 μg/mg; CV 3.8%). All the remaining laboratories determined the 5α-cholestanol to cholesterol ratio within a ± 15% range of the corrected mean value.

In the second survey part seven participants were included with calculated results for sample A (2.10 ± 1.41 μg/mg; CV 67.1%) and B (2.41 ± 2.33 μg/mg; CV 96.5%). None of the participants determined 5α-cholestanol to cholesterol ratio within a ± 15% range of the mean value of all participants and two participants were identified as outliers (Fig. 2 A2, a and b). The new corrected mean value for the remaining five laboratories were calculated for A (1.92 ± 0.49 μg/mg; CV 25.6%) and B (1.69 ± 0.17 μg/mg; CV 10.1%). Three of the remaining five laboratories determined the 5α-cholestanol to cholesterol ratio within a ± 15% range of the corrected mean value.

#### 3.6.2 Lathosterol to cholesterol

In the first survey part seven participants were included with calculated results for sample A (1.48 ± 1.32 μg/mg; CV 89.1%) and B (1.41 ± 1.17 μg/mg; CV 82.8%). Two of the participants determined lathosterol to cholesterol ratio within a ± 15% range of the mean value of all participants and three participants were identified as outliers (Fig. 2 B1, a to c). The new corrected mean value for the remaining four laboratories were calculated for A (1.17 ± 0.41 μg/mg; CV 35.4%) and B (1.16 ± 0.39 μg/mg; CV 33.7%). None of the laboratories determined the lathosterol to cholesterol ratio within a ± 15% range of the corrected mean value.

In the second survey part eleven participants were included with calculated results for sample A (1.28 ± 0.40 μg/mg; CV 31.4%) and B (1.15 ± 0.47 μg/mg; CV 40.5%). Five of the participants determined lathosterol to cholesterol ratio within a ± 15% range of the mean value of all participants and three participants were identified as outliers (Fig. 2 B2, a to c). The new corrected mean value for the remaining five laboratories were calculated for A (1.28 ± 0.15 μg/mg; CV 11.7%) and B (1.25 ± 0.15 μg/mg; CV 12.2%). Six of the remaining eight laboratories determined the lathosterol to cholesterol ratio within a ± 15% range of the corrected mean value.

#### 3.6.3 Campesterol to cholesterol

In the first survey part eight participants were included with calculated results for sample A (3.94 ± 2.10 μg/mg; CV 53.2%) and B (4.20 ± 2.06 μg/mg; CV 49.0%). None of the participants determined campesterol to cholesterol ratio within a ± 15% range of the mean value of all participants and two participants were identified as outliers (Fig. 2 C1, a and b). The new corrected mean value for the remaining six laboratories were calculated for A (3.56 ± 0.99 μg/mg; CV 27.9%) and B (3.81 ± 1.17 μg/mg; CV 30.6%). Two laboratories determined the campesterol to cholesterol ratio within a ± 15% range of the corrected mean value.

In the second survey part thirteen participants were included with calculated results for sample A (3.71 ± 1.86 μg/mg; CV 50.1%) and B (3.70 ± 1.98 μg/mg; CV 53.5%). One of the participants determined campesterol to cholesterol ratio within a ± 15% range of the mean value of all participants and five participants were identified as outliers (Fig. 2 C2, a and e). The new corrected mean value for the remaining eight laboratories were calculated for A (3.24 ± 1.06 μg/mg; CV 32.7%) and B (3.24 ± 1.01 μg/mg; CV 31.3%). One of the remaining eight laboratories determined the campesterol to cholesterol ratio within a ± 15% range of the corrected mean value.

#### 3.6.4 Lathosterol to campesterol

In the first survey part twelve participants were included with calculated results for sample A (0.45 ± 0.49 μg/μg; CV 109.6%) and B (0.52 ± 0.57 μg/μg; CV 109.2%). One of the participants determined lathosterol to campesterol ratio within a ± 15% range of the mean value of all participants and six participants were identified as outliers (Fig. 2 D1, a to f). The new corrected mean value for the remaining six laboratories were calculated for A (0.35 ± 0.09 μg/μg; CV 26.2%) and B (0.33 ± 0.08 μg/μg; CV 24.1%). One laboratory determined the lathosterol to campesterol ratio within a ± 15% range of the corrected mean value.

In the second survey part fifteen participants were included with calculated results for sample A (0.55 ± 0.28 μg/μg; CV 51.5%) and B (0.54 ± 0.30 μg/μg; CV 56.4%). Seven of the participants determined lathosterol to campesterol ratio within a ± 15% range of the mean value of all participants and two participants were identified as outliers (Fig. 2 D1, a and b). The new corrected mean value for the remaining eight laboratories were calculated for A (0.50 ± 0.10 μg/μg; CV 20.1%) and B (0.50 ± 0.09 μg/μg; CV 18.7%). Seven of the remaining eleven laboratories determined the lathosterol to campesterol ratio within a ± 15% range of the corrected mean value.

The coefficient of variation for the average value for the ratio of 5α-cholestanol, lathosterol- and campesterol to cholesterol were calculated to be between 31.4% and 96.5%, respectively. The coefficient of variation for the average value for the ratio of lathosterol to campesterol were calculated between 51.5% to 109.2%. Here is to notice that the ratio of lathosterol to campesterol shows even higher variations probably because of the analytical challenges. It is noteworthy that this survey revealed a high variation for ratios of NCS to cholesterol which prevents definition of resilient threshold values.

## 4 Common discussion

Unlikely to the other NCS assays, a target value for cholesterol could be reported in the present survey. For NCS assays the reported values could only be compared to the mean of values obtained from all participants or to a corrected mean value after removal of defined outlier. Thus, the true NCS analyte concentration could not be referred with certainty in any of these assays. Absolute reference methods have been developed for some analytes of diagnostic importance but for most analytes there is no “golden method” and we are thus restricted to compare results between different laboratories [9,10].

However, with the collected survey data on hand it is not possible to sufficiently evaluate the best chromatographic method for cholesterol and NCS determination in plasma or serum. It can be generally considered that the quality of the present type of cholesterol and NCS analysis is directly dependent upon the skill and experience of the analyst, the limitations of the analysis method, the sample quality and sampling itself, as well as on the quality of the calibration material and used internal standards. Within the survey six different methods were used whereby all measurements dependent upon a critical chromatographic step, either gas- or liquid chromatography. Due to higher chromatographic efficiency and the reduced influence of matrix effects on ionisation and signal suppression, one would expect less analytical problems with gas chromatography compared to liquid chromatography. As ideal standard for cholesterol and NCS analysis it can be expected that the use of the same deuterium or ^13^C isotope labelled molecule will result in the most precise mass spectrometric analysis. In respect to that it is to mention that the internationally accepted reference method for cholesterol is based on isotope-dilution mass-spectrometry combined with GC [8,9,[11], [12], [13]].

The use of common quantified calibration material slightly improved the variation of reported results even since individual laboratories and the number of participants is different in the two survey parts. Here, three points should be emphasized: First of all, commercially available standards do not always have the required purity of 99.0% needed. Quality and purity of standard materials used should always be controlled if possible. In addition to this, the weighing of small milligram amounts is often challenging and is prone to generate errors. To avoid these effects of impurities and weighing, we recommend to quantify the cholesterol and NCS calibration solutions by GC-FID (5α-cholestanol) before use. Lastly, calibration solutions independent if self-prepared or commercially sourced have a limited bench stability. Using the same solution over years might alter internal analyte concentrations. Therefore, used stock solutions should be newly prepared or re-quantified after a certain usage time by the above described method and old stock solutions should be analysed alongside the new for a short overlapping period.

## 5 Conclusions

We found surprisingly high variations in cholesterol and NCS concentrations obtained from analytical assays based on chromatographic separation. The participating laboratories specialized in lipid analysis reported astoundingly strong differences in the concentrations of neutral sterols in both survey parts. However, the use of common calibration materials slightly improved the variations in the reported concentrations, whereby still high variations were obtained in the second interventional survey part. In glance to this situation the evaluation of normal plasma or serum reference concentrations of neutral sterols is very challenging and nearly impossible. Furthermore, this survey gives evidence for the urgent need for quality control programs with interchange of samples between different laboratories in order to harmonize the chromatographic analysis of cholesterol and NCS from serum or plasma samples. In the future we intent to provide following ring trials for the quantification of cholesterol, oxidized cholesterols and neutral sterols based on chromatographic separation techniques and mass spectrometric or flame ionization detection methods.

## Fundings

We gratefully acknowledge funding for materials, statistics, and travel expenses to the Foundation for Pathobiochemistry and Molecular Diagnostics, German Society of Clinical Chemistry and Laboratory Medicine, Germany. The work in University of Valencia was financed by CICYT-FEDER (AGL2008−02591-C02-01) and MINECO-FEDER (AGL2012−39503-C02-01). The work at Universitario Miguel Servet, Zaragoza, Spain was funded by Fondo de Investigación Sanitaria (FIS; PI15/01983). Work in Swansea was supported by the UK Biotechnology and Biological Sciences Research Council (BBSRC, grant numbers BB/I001735/1 and BB/L001942/1).

## References

[1] Björkhem I., Miettinen T., Reihner E., Ewerth S., Angelin B., Einarsson K. (1987). Correlation between serum levels of some cholesterol precursors and activity of HMG-CoA reductase in human liver. J. Lipid Res..

[2] Miettinen T.A., Tilvis R.S., Kesaniemi Y.A. (1989). Serum cholestanol and plant sterol levels in relation to cholesterol metabolism in middle-aged men. Metabolism.

[3] Tilvis R.S., Miettinen T.A. (1986). Serum plant sterols and their relation to cholesterol absorption. Am. J. Clin. Nutr..

[4] Miettinen T.A., Tilvis R.S., Kesaniemi Y.A. (1990). Serum plant sterols and cholesterol precursors reflect cholesterol absorption and synthesis in volunteers of a randomly selected male population. Am. J. Epidemiol..

[5] Mackay D., Jones P.J. (2011). Evaluation of methods for the determination of cholesterol absorption and synthesis in humans. Atherosclerosis.

[6] Mackay D.S., Jones P.J. (2012). Plasma noncholesterol sterols: current uses, potential and need for standardization. Curr. Opin. Lipidol..

[7] Mackay D.S., Jones P.J., Myrie S.B., Plat J., Lütjohann D. (2014). Methodological considerations for the harmonization of non-cholesterol sterol bio-analysis. J. Chromatogr. B Anal. Technol. Biomed. Life Sci..

[8] Bundesärztekammer (2014). Richtlinie der Bundesärztekammer zur Qualitätssicherung laboratoriumsmedizinischer Untersuchungen. Deutsches Ärzteblatt.

[9] Gylling H., Simonen P., Kaipiainen L., Wester I. (2018). Methodological aspects of phytosterol measurements in biological samples. Curr. Med. Chem..

[10] Srigley C.T., Hansen S.L., Smith S.A., Abraham A., Railey E., Chen X., Chooi S.H., Clement L.M., Dao M., Kia A.R.F., Mitchell B., Mogla M., Ortiz J.A.R., Persons K., von Kries E., Ware G., Wubben J., Cantrill R. (2018). Sterols and stanols in foods and dietary supplements containing added phytosterols: a collaborative study. J. Am. Oil Chem. Soc..

[11] Edwards S.H., Kimberly M.M., Pyatt S.D., Stribling S.L., Dobbin K.D., Myers G.L. (2011). Proposed serum cholesterol reference measurement procedure by gas chromatography-isotope dilution mass spectrometry. Clin. Chem..

[12] Björkhem I., Blomstrand R., Eriksson S., Falk O., Kallner A., Svensson L., Ohman G. (1980). Use of isotope dilution--mass spectrometry for accuracy control of different routine methods used in clinical chemistry. Scand. J. Clin. Lab. Invest..

[13] Björkhem I., Blomstrand R., Svensson L. (1974). Serum cholesterol determination by mass fragmentography. Clin. Chim. Acta.

